# Dynamics of the Gut Microbiota and Faecal and Serum Metabolomes during Pregnancy—A Longitudinal Study

**DOI:** 10.3390/nu16040483

**Published:** 2024-02-07

**Authors:** Ruili Pan, Min Guo, Ying Chen, Guopeng Lin, Peijun Tian, Linlin Wang, Jianxin Zhao, Wei Chen, Gang Wang

**Affiliations:** 1 State Key Laboratory of Food Science and Resources, Jiangnan University, Wuxi 214122, China; ruili_pan1222@163.com (R.P.); guomin@jiangnan.edu.cn (M.G.); manju_ying@163.com (Y.C.); 7200112079@stu.jiangnan.edu.cn (G.L.); pjtian@jiangnan.edu.cn (P.T.); zhaojianxin@jiangnan.edu.cn (J.Z.); chenwei66@jiangnan.edu.cn (W.C.); wanggang@jiangnan.edu.cn (G.W.); 2 School of Food Science and Technology, Jiangnan University, Wuxi 214122, China; 3 National Engineering Research Center for Functional Food, Jiangnan University, Wuxi 214122, China; 4 (Yangzhou) Institute of Food Biotechnology, Jiangnan University, Yangzhou 225004, China

**Keywords:** pregnancy, postpartum period, gastrointestinal microbiome

## Abstract

Normal pregnancy involves numerous physiological changes, including changes in hormone levels, immune responses, and metabolism. Although several studies have shown that the gut microbiota may have an important role in the progression of pregnancy, these findings have been inconsistent, and the relationship between the gut microbiota and metabolites that change dynamically during and after pregnancy remains to be clarified. In this longitudinal study, we comprehensively profiled the temporal dynamics of the gut microbiota, *Bifidobacterium* communities, and serum and faecal metabolomes of 31 women during their pregnancies and postpartum periods. The microbial composition changed as gestation progressed, with the pregnancy and postpartum periods exhibiting distinct bacterial community characteristics, including significant alterations in the genera of the Lachnospiraceae or Ruminococcaceae families, especially the *Lachnospiraceae FCS020 group* and *Ruminococcaceae UCG-003*. Metabolic dynamics, characterised by changes in nutrients important for fetal growth (e.g., docosatrienoic acid), anti-inflammatory metabolites (e.g., trans-3-indoleacrylic acid), and steroid hormones (e.g., progesterone), were observed in both serum and faecal samples during pregnancy. Moreover, a complex correlation was identified between the pregnancy-related microbiota and metabolites, with *Ruminococcus1* and *Ruminococcaceae UCG-013* making important contributions to changes in faecal and serum metabolites, respectively. Overall, a highly coordinated microbiota–metabolite regulatory network may underlie the pregnancy process. These findings provide a foundation for enhancing our understanding of the molecular processes occurring during the progression of pregnancy, thereby contributing to nutrition and health management during this period.

## 1. Introduction

Normal pregnancy is a unique biological process involving simultaneous changes in various physiological systems to support fetal development [1]. Critical metabolic changes, resembling those occurring in metabolic syndrome, especially insulin resistance and dyslipidaemia, occur during pregnancy [2]. The concentrations of key nutrients, such as circulating lipids and amino acids, increase considerably with advancing gestation [3]. These changes are essential for fetal growth and the accumulation of energy reserves for breastmilk production. Additionally, maternal immunity undergoes complex adaptive changes: A certain degree of immunosuppression is required to accommodate the growing fetus, whose own immune system is developing, while robust immunity must be maintained to protect both the mother and the fetus from infection [4].

Notably, the gut microbiome plays a critical role in health, influencing nutrient acquisition [5], immune programming [6], and metabolic homeostasis [7]. Faecal microbiota transplant (FMT) of third trimester (T3) samples from pregnant women have induced weight gain, insulin resistance, and inflammatory response [8]. Thus, the microbiome may both influence and be influenced by the abovementioned pregnancy-related changes. Although several longitudinal studies have reported temporal changes in microbial composition during pregnancy, conflicting results exist. The composition of intestinal flora has been noted to change significantly from the first trimesters (T1 to T3), with reduced richness [8,9]. However, DiGiulio et al. found no dramatic remodelling of microbial composition over the course of pregnancy [10]. Given the potential role of microbial disturbance in pregnancy complications [11], understanding the temporal dynamics of the gut microbiota in the context of a healthy pregnancy is crucial.

In addition to the gut microbiota, microbiota-related metabolites interact with the host and transmit intestinal signals to the entire system, which may be precisely programmed to maintain a normal pregnancy [12]. Metabolism during normal pregnancy is a dynamic and precisely programmed process. Compared with the non-pregnancy period, pregnancy triggers substantial metabolic changes, such as alteration in the serum levels of fatty acids and amino acids, in women [13]. Liang et al. systematically characterized the serum metabolomic profile throughout pregnancy with the weekly sampling of maternal serum and found that functional metabolites, especially steroid hormones and long-chain fatty acids, were changed over the gestational period [14]. Most metabolite changes rapidly returned to pre-pregnancy levels after childbirth [14]. The faecal metabolome can help clarify the influence and regulatory mechanism of intestinal microorganisms on host metabolism [15]. However, little is known about the temporal characteristics of faecal metabolites in a healthy pregnancy. Considering the impact of microbiota–metabolite cross-talk on the host, it is important to simultaneously characterise how microbial, serum, and faecal metabolic signatures longitudinally alter and interact during and after a healthy pregnancy, which helps to fully understand how the pregnancy is regulated.

In this longitudinal study, we comprehensively characterised the dynamic remodelling process of the gut microbiota and serum and faecal metabolomes. Integrated association analysis was performed to evaluate significant microbiota–metabolite interactions over the course of a normal pregnancy and 1-week postpartum. These findings may provide novel insights into maternal physiological changes with advancing gestation and subsequent pregnancy health management.

## 2. Materials and Methods

### 2.1. Pregnancy Cohort

The samples used in this study originated from a prospective nested case–control study that commenced from October 2021 to October 2022 and involved women in the early stage of pregnancy, with follow-up to the postpartum period. The operation protocol was approved by the Research Ethics Committee of the Wuxi Xishan People’s Hospital (Approval Code, xs2020ky010) and was registered with the Chinese Clinical Trial Registry (No. ChiCTR2100052265). All participants who agreed to participate in this study provided written informed consent prior to their enrolment.

Specifically, pregnant women in their first trimester, aged 18–40 years, who underwent prenatal examinations at Xishan People’s Hospital were invited to participate. Pregnant women were excluded if they (1) had been diagnosed with a systemic, endocrine, or urinary system disease or another chronic medical condition; (2) experienced gestational diseases during pregnancy, including hyperemesis gravidarum, gestational diabetes, pre-eclampsia, or intrahepatic cholestasis of pregnancy; or (3) used antibiotics, probiotics, or medication affecting the gut microbiome during pregnancy. Importantly, the pregnant women included in this study lived within 30 km of the study hospital, did not leave the city of Wuxi, and maintained consistent dietary patterns throughout their pregnancies.

### 2.2. Sample Collection

In each trimester of pregnancy and postpartum, serum samples were collected by professional medical staff after fasting and stored in −80 °C refrigerators after centrifugation and equal volume packaging. Additionally, faecal samples were collected in each trimester of pregnancy and the postpartum period using sterile containers with ice boxes and immediately transferred to −80 °C freezers.

### 2.3. Clinical Measurements and Blood Index Evaluation

The general characteristics of all participants were recorded at recruitment, and clinical data were obtained from their medical records. Gestational weight gain was determined by calculating the difference between the weight at each trimester and the pre-pregnancy weight. Routine blood examinations in each trimester were conducted using an XN-Series automated haematology analyser (SYSMEX, Kobe, Japan).

### 2.4. Faecal DNA Extraction, 16S rRNA Sequencing, and GroEL Gene Sequencing

The sequencing of 124 faecal samples was performed on a MiSeq platform (Illumina, San Diego, CA, USA) following established procedures, including the following experimental steps [16]: (1) genomic DNA was extracted using a FastDNA Spin Kit (MP Biomedical, Irvine, CA, USA); (2) the V3–V4 region was amplified by polymerase chain reaction (PCR) with a universal primer pair (341F/806R); (3) the amplified products were purified and quantified using a Qubit dsDNA Assay Kit (Life Technologies, Invitrogen, Carlsbad, CA, USA); (4) the purified PCR products were pooled at equal concentrations; and (5) paired-end sequenced was performed on a MiSeq platform.

The process for sequencing the *GroEL* gene of *Bifidobacterium* [17] was similar to the process for standard *16S rRNA* sequencing but with different primers and PCR amplification conditions, as described previously [18].

### 2.5. Sequence Data Processing and Bioinformatic Analysis

Sequence data were processed using QIIME2 [19]. The Ribosomal Database Project Classifier and *Bifidobacterium* GroEL Database were used for taxonomical classification of the *16S rRNA* and *GroEL* sequencing data. Additionally, α-diversity (within-sample diversity) analysis involving the Shannon and Chao1 indices was performed based on the relative abundance at the genus level. The β-diversity (between-sample diversity) was indicated by the genus-level Bray–Curtis distance and was visualised through principal coordinates analysis (PCoA). Additionally, the differences in β-diversity between groups were assessed through permutational multivariate analysis of variance involving 999 permutations. Taxonomic biomarkers of different trimesters were determined using the Kruskal–Wallis test and linear discriminant analysis effect size (LEfSe). Furthermore, the gut microbiome function was predicted using the Phylogenetic Investigation of Communities by Reconstruction of Unobserved States2 (PICRUSt2, https://github.com/picrust/picrust2, accessed on 6 February 2023) software [20].

### 2.6. Untargeted Metabolomics for Faecal and Serum Samples

Metabolites were extracted from stool samples following established methods with some modifications [21]. Aliquots of lyophilised faecal samples were placed in centrifuge tubes containing grinding beads and treated with a pre-cooled extraction solvent (ultra-pure water: methanol: acetonitrile: = 1:2:2, [*v*/*v*]), followed by homogenisation (65 Hz, 30 s, three times). After protein precipitation at −20 °C for 1 h, the mixture was centrifuged at 15,000× *g* for 10 min to collect the supernatants for concentration. Subsequently, the metabolic extracts were dissolved in 50% (*v*/*v*) acetonitrile and subjected to ultraperformance liquid chromatography with tandem mass spectrometry (UPLC-MS) detection. The preparation of serum samples was similar to the method used for stool samples, except that the extraction solvent used was methanol. Notably, a quality control (QC) sample was prepared by mixing each sample equally. The QC sample was regularly injected to monitor the stability of untargeted metabolomics analysis and signal intensity [22].

Untargeted metabolomic profiling was conducted using an UItiMate 3000 UPLC system connected to a high-resolution Q Exactive Mass Spectrometer and UPLC High-Strength Silica T3 column (2.1 × 100 mm, 1.8 µm; Waters, Milford, MA, USA). The UPLC-MS operating conditions were set in accordance with published protocols [21].

Peak alignment, peak picking, and peak identification were performed, and the retention time (RT) and *m*/*z* data for each peak were obtained using Compound Discovery 3.3 (CD 3.3, Thermo Fisher Scientific, Waltham, MA, USA) following an untargeted metabolomic workflow [21]. Only metabolomic features appearing in >50% of the QC samples and showing <30% relative standard deviation in the QC samples were retained for further analysis. Notably, MS signal drift with time was independently corrected by locally estimated scatterplot smoothing normalisation. Metabolite annotations were performed using mzVault, mzCloud, ChemSpider, and the Human Metabolome Database. Only metabolite annotations with mzCloud MS/MS spectral match scores ≥ 70 were considered credible [23].

The obtained metabolite data were imported into MetaboAnalyst 5.0 (https://www.metaboanalyst.ca/, accessed on 8 April 2023) for further analysis [24]. Spearman’s correlation analysis and principal component analysis plots for all QC data were used to examine the stability of the metabolic data and check the run quality. Orthogonal partial least squares discriminant analysis (OPLS-DA) was performed to characterise the overall distribution within the groups and the degree of difference between different trimesters and the postpartum period. Furthermore, discriminative metabolites were identified based on the criteria of a variable importance plot (VIP) score > 1.0 and *p* < 0.05 [25]. Pathway enrichment analysis was performed for distinctive markers using the Kyoto Encyclopedia of Genes and Genomes database.

### 2.7. Targeted Metabolomics of Short-Chain Fatty Acids (SCFAs) by Gas Chromatography–Mass Spectrometry (GC-MS)

The extraction and quantification of faecal SCFAs were performed with reference to reported protocols [26]. Briefly, 30 mg of freeze-dried faecal samples were dissolved with sodium chloride and acidified with sulphuric acid, followed by extraction with diethyl ether. The SCFAs in the resulting extracts were identified using GC-MS. Raw GC-MS data were analysed using the Xcalibur 4.7 software (Thermo Fisher Scientific), followed by manual verification of the RT.

### 2.8. Statistical Analysis

Various software applications, including Prism version 8.0.2 (GraphPad, San Diego, CA, USA), SPSS version 22 (IBM, Armonk, NY, USA), and R 4.0.3 (https://www.r-project.org/, accessed on 3 December 2022), were used for statistical analysis. The Shapiro–Wilk test was performed to assess normal distribution. For normally distributed data, Student’s *t*-test or one-way analysis of variance with the Holm–Sidak test was performed. For nonparametric data, a Wilcoxon rank-sum test, Fisher’s exact test, or Kruskal–Wallis test was conducted, followed by Dunn’s test or Welch’s *t*-test. Data are presented as mean values ± standard errors of mean or the median ± interquartile range. *p* < 0.05 was considered to represent statistical significance. Spearman’s correlation analysis was performed based on differential microbiota and metabolites between different trimesters, with visualisations obtained using Gephi (*p* < 0.05).

## 3. Results

### 3.1. Participants’ Clinical Characteristics

Thirty-one pregnant women were included in this longitudinal study. Each participant provided faecal and serum samples during T1 (10.9 ± 0.33 weeks), the second trimester (T2) (23.0 ± 0.47 weeks), T3 (35.5 ± 0.35 weeks), and the postpartum period (day 5.2 ± 0.18 after delivery) (Table 1). All women had normal body mass indexes in the pre-pregnancy stage and had no diagnosed pregnancy complications. The weight, metabolism-related indicators (e.g., triglycerides), liver-function-related indicators (e.g., alkaline phosphatase), and kidney-function-related indicators (e.g., cystatin C) were found to change significantly with advancing gestation.

### 3.2. Dramatic Changes in the Gut Microbiota with Advancing Gestation

**Figure 1:**
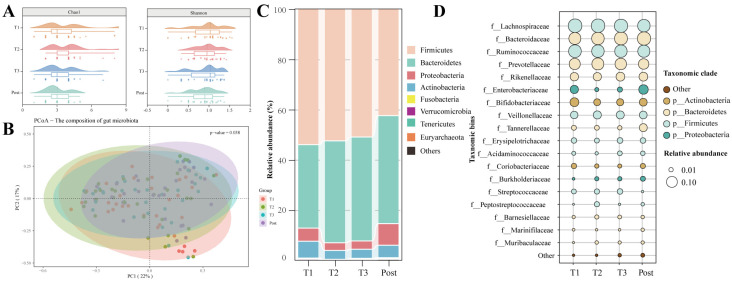
Temporal dynamics of gut microbiota composition during pregnancy. (**A**) Alpha diversity analysis indicated by Chao1 and Shannon indices. (**B**) Beta diversity characterised by a Bray–Curtis analysis. (**C**) Microbial distribution at the phylum level. (**D**) Microbial distribution at the family level. T1: first trimester; T2: second trimester; T3: third trimester (T3); Post: postpartum.

To comprehensively examine the compositional dynamics of the microbiota during normal gestation, *16S rRNA* sequencing of faecal samples, collected longitudinally in each trimester and the postpartum period, was performed. No difference was observed in the α-diversity (i.e., Chao1 and Shannon indices), as indicated in Figure 1A. However, the PCoA based on Bray–Curtis distances suggested distinct variations in the overall composition of the gut microbiome as gestation progressed (*p* = 0.038) (Figure 1B).

**Figure 2:**
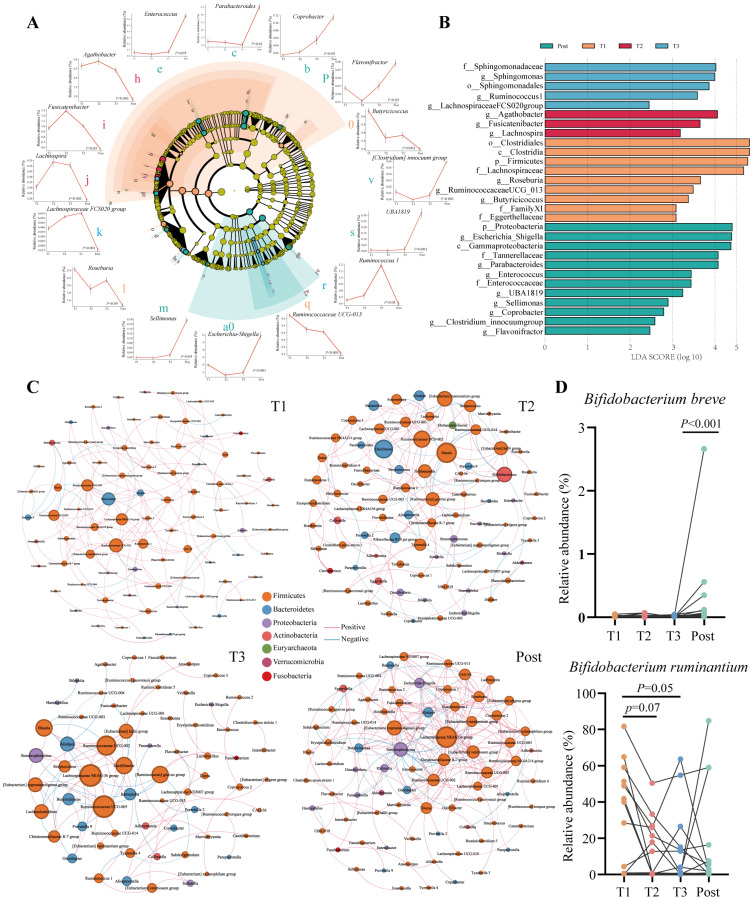
Changes in the gut microbiota at the genus level as pregnancy progressed. (**A**) Cladogram of the characterised microbiota and the average levels of the important significant microbiota changes as gestation progressed. The letters correspond to the names of the significant microbiota marked in the line chart. The colors of the letters correspond to the colors in the cladogram. (**B**) Distribution histogram of the characterised microbiota based on LDA. (**C**) Co-occurrence network graph of gut microbiota in T1, T2, and T3 and postpartum at the genus level. Node size indicates the degree of corresponding factors. Only *p*-values < 0.05 and |r| ≥ 0.5 are displayed in the network. (**D**) The relative abundance of *Bifidobacterium breve* and *Bifidobacterium ruminantium.* T1: first trimester; T2: second trimester; T3: third trimester (T3); Post: postpartum.

Temporal changes in the gut microbial profiles during pregnancy and the postpartum period were observed from the phylum to genus levels. At the phylum level, the relative abundance of the dominant bacteria, Bacteroidetes, increased from T1 to T2, and the abundances of Proteobacteria and Actinobacteria varied with advancing gestation (Figure 1C). At the family level, temporal changes in the relative abundance of Enterobacteriaceae were observed during and after pregnancy, and Bifidobacteriaceae was noted to be enriched in T1 compared with T3 (Figure 1D). At the genus level, differential microbiota characterising each trimester and the postpartum period were identified by Lefse analysis (Figure 2A,B). *Butyricicoccus*, *Roseburia*, and *Ruminococcaceae UCG-013* were predominant in T1, while T2 was characterised by genera belonging to the Lachnospiraceae family, including *Agathobacter*, *Fusicatenibacter*, and *Lachnospira*. *Sphingomonas, Ruminococcus1*, and the *Lachnospiraceae FCS020* group were abundant in T3. Compared with pregnant women, women in the immediate postpartum period displayed an abundance of pathogenic bacteria, especially *Escherichia_Shigella*, *Enterococcus,* and *UBA1819*. Taxonomic biomarkers presented different trends as pregnancy progressed. The trends were maintained for some of the bacteria but reversed in others during the postpartum period (Figure 2A). For example, the relative abundance of *Ruminococcaceae UCG-013* progressively decreased with advancing gestation until the postpartum period, while the levels of *Ruminococcus1* gradually increased as pregnancy progressed but decreased after delivery. These findings suggested that the gut microbiota exhibits temporal changes as pregnancy progresses.

### 3.3. Symbiotic Network Analysis of the Gut Microbiota in Pregnant Women

The dynamic balance of the intestinal ecosystem is maintained by the interactions of intestinal microorganisms. Hence, we performed Spearman’s correlation analysis and constructed symbiotic networks for the top 100 most abundant genera to reveal the interactions between gut microbes and identify the core genera in each trimester and postpartum (Figure 2C). The gut microbiota in T3 has the fewest microbiota interactions compared with other trimesters. In T1, the dominant microbiota in the network were *Ruminococcaceae UCG-002*, *Butyricimonas*, and the *Lachnospira*ceae *NK4A136 group*. *Blautia*, *Odoribacter*, and *Ruminococcaceae UCG-002* occupied an important advantage in T2. The *Lachnospira*ceae *NK4A136 group* was the main contributor to the co-occurrence network both in T3 and postpartum. The symbiotic correlations between the gut microbiota showed time-dependent changes during pregnancy.

### 3.4. Minimal Changes in Bifidobacterium Communities at the Species Level with Advancing Gestation

Considering the significance of *Bifidobacterium* in early life development, we examined *Bifidobacterium* communities at the species level. High-throughput sequencing of *GroEL* regions was performed for faecal samples collected in T1, T2, and T3 and the postpartum period, and 28 species of *Bifidobacterium* were detected. However, no significant temporal changes were observed in the *Bifidobacterium* structures or signatures over the course of normal pregnancy (Figure S1A). Only certain *Bifidobacterium* species exhibited subtle changes during pregnancy (Figure 2D). For example, the abundance of *Bifidobacterium breve* significantly increased from T3 to the postpartum period, while the abundance of *Bifidobacterium ruminantium* decreased from T1 to T2 (*p* = 0.05). These findings suggested minimal impact on *Bifidobacterium* communities during pregnancy.

### 3.5. Changes in Functional Pathways of Gut Microbiota during Pregnancy

We conducted a PICRUSt2 analysis to predict functional pathways in the gut microbiota. The differential functional pathways pertained to immune-related signalling pathways (e.g., the p53 and FoxO signalling pathways), metabolism-related signalling pathways (the insulin signalling pathway, the apelin signalling pathway, and energy metabolism), and signalling pathways related to fatty acids or amino acids (e.g., tryptophan metabolism and fatty acid degradation) (Figure S1B).

### 3.6. Temporal Changes in Faecal and Serum Metabolomic Profiles during Pregnancy and the Postpartum Period

**Figure 3:**
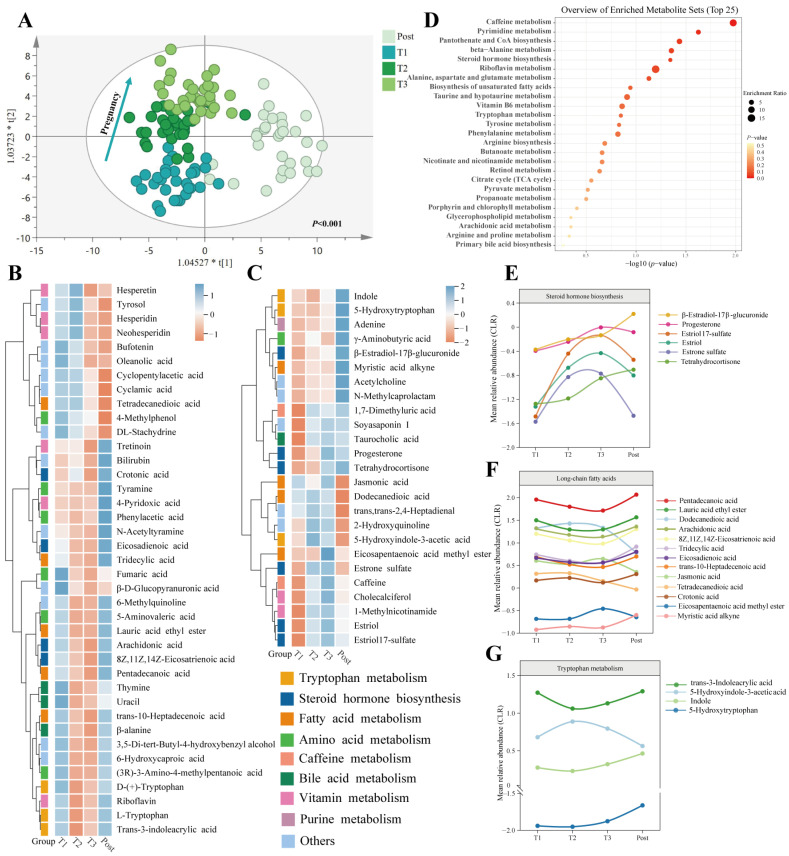
Temporal changes in faecal metabolites during pregnancy. (**A**) OPLS-DA score plot. Heatmap exhibits the metabolite signal intensity averaged across pregnant women, showing important significant metabolites decreased (**B**) and increased (**C**) by the end of pregnancy. (**D**) Metabolite set enrichment analysis of pregnancy-related metabolites based on the KEGG database. The average levels of the metabolite change with advancing gestation in the clusters of steroid hormone biosynthesis (**E**), long-chain fatty acids (**F**), and tryptophan metabolism (**G**). The y-axis shows CLR-transformed metabolite concentrations. T1: first trimester; T2: second trimester; T3: third trimester (T3); Post: postpartum.

**Figure 4:**
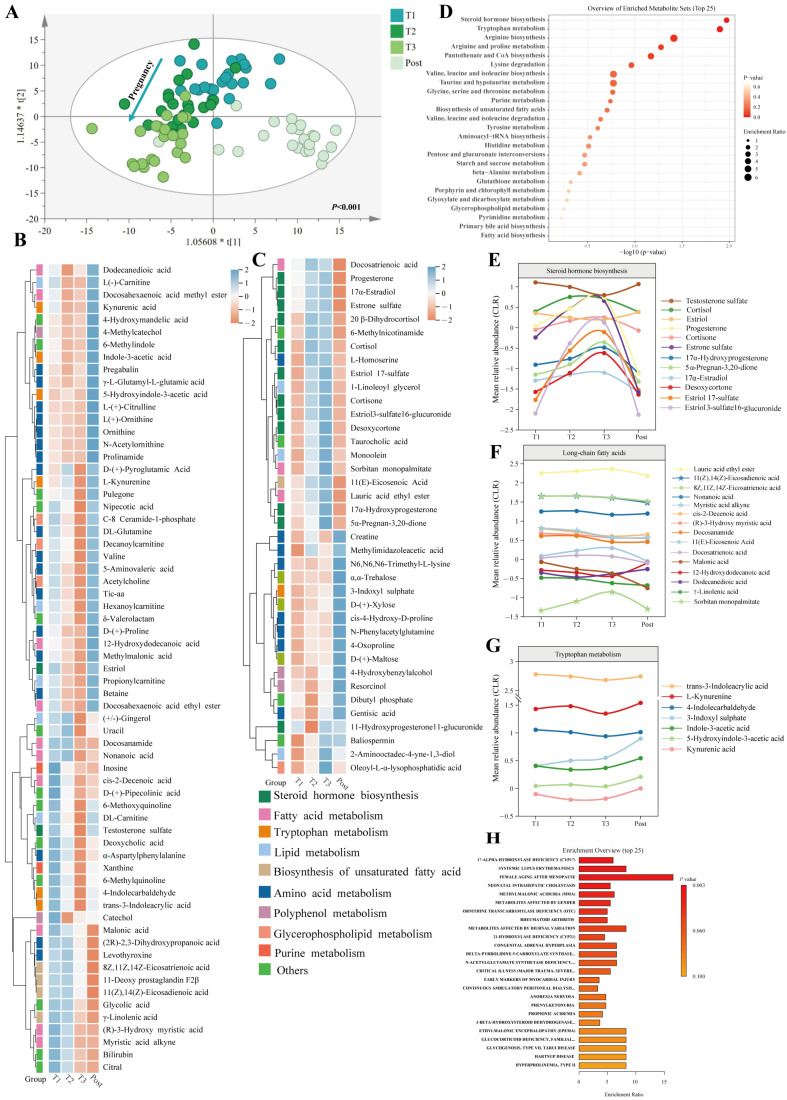
Temporal changes in serum metabolites during pregnancy. (**A**) OPLS-DA score plot. Heatmap exhibits the metabolite signal intensity averaged across pregnant women, showing important significant metabolites decreased (**B**) and increased (**C**) by the end of pregnancy. (**D**) Metabolite set enrichment analysis of pregnancy-related metabolites based on the KEGG database. The average levels of the metabolite change with advancing gestation in the clusters of steroid hormone biosynthesis (**E**), long-chain fatty acids (**F**), and tryptophan metabolism (**G**). The y-axis shows CLR-transformed metabolite concentrations. (**H**) Human disease states associated with pregnancy-related metabolites. T1: first trimester; T2: second trimester; T3: third trimester (T3); Post: postpartum.

Gut-microbiota-related metabolites interact with the host to regulate host homeostasis. Untargeted metabolomics analysis was performed on faecal and serum samples to clarify the functional role of the gut microbiota as gestation progresses. A total of 1026 metabolites, including 542 serum metabolites and 484 faecal metabolites, exhibited MS/MS spectral best-match scores greater than or equal to 70 in the mzCloud database. In faecal-positive (ESI^+^) and -negative electrospray ionisation (ESI^−^) samples, as well as serum ESI^+^ and ESI^−^ samples, the QC samples were tightly clustered (Figure S2A–D). Additionally, Spearman’s correlation coefficients of the ESI^+^ and ESI^−^ QC data for both faecal and serum samples were high, reflecting the stability and accuracy of the untargeted metabolomics data (Figure S2E–H). OPLS-DA analysis revealed that the dynamic distributions of metabolomic data for both faecal and serum samples changed significantly as gestation progressed (*p* < 0.001), and the overall faecal and serum metabolic signatures were different before and after childbirth (Figure 3A and Figure 4A).

Among the faecal metabolites, 98 showed considerable alterations with advancing gestation based on a combination of the statistical parameters of univariate (*p* < 0.05) and multivariate analyses (VIP > 1). Existing structural and biological information was used to categorise the metabolites with the most notable changes to clarify the functional groups (Figure 3B,C). Metabolic pathways showing changes corresponded to caffeine metabolism, steroid hormone biosynthesis, pyrimidine metabolism, the biosynthesis of unsaturated fatty acids, and amino acid metabolism (including tryptophan and beta-alanine) pathways during pregnancy and the postpartum period (Figure 3D). Significant metabolites related to steroid hormone biosynthesis, including estriol, progesterone, and estriol 17-sulfate, consistently increased during pregnancy, with most decreasing sharply after delivery (Figure 3E). Compounds related to fatty acid metabolism exhibited distinct variation trends. For example, the concentrations of pentadecanoic acid and tridecylic acid steadily decreased during pregnancy and increased after delivery, while dodecanedioic acid showed the opposite trend (Figure 3F). Notably, the concentrations of unsaturated fatty acids, including arachidonic acid, eicosadienoic acid, and 8Z,11Z,14Z-eicosatrienoic acid, gradually decreased as gestation progressed and then increased in the postpartum period (Figure 3F). The metabolites related to tryptophan metabolism exhibited intricate temporal changes during pregnancy. For example, the concentrations of trans-3-indoleacrylic acid and indole decreased from T1 to T2 and then steadily increased until childbirth, while 5-hydroxyindole-3-acetic acid showed the opposite trend. Interestingly, important neurotransmitters, such as acetylcholine and γ-aminobutyric acid, presented temporal variations during pregnancy and the postpartum period. Overall, the functional faecal metabolites showed significant and programmed changes as gestation progressed.

In serum samples, 105 differential serum metabolites were identified and categorised based on existing structural and biological information (Figure 4B,C). Similar to the faecal metabolome, these differential serum metabolites were mainly enriched in pathways associated with steroid hormone biosynthesis, tryptophan metabolism, and the biosynthesis of unsaturated fatty acids (Figure 4D). The number of changed metabolites related to steroid hormone biosynthesis in serum samples exceeded the number in stool samples. Most serum steroid hormone concentrations followed the same trend as those in faeces, gradually increasing during pregnancy but decreasing soon after delivery, except for estriol (Figure 4E). The concentrations of most unsaturated fatty acids continued to decline during pregnancy, following the trend observed in stool samples (Figure 4F). All three major pathways of tryptophan metabolism, involving serotonin, kynurenine, and indole derivatives, were altered (Figure 4G). Indole-3-acetic acid and kynurenic acid concentrations decreased from T1 to T2 and then generally increased until delivery. In contrast, the concentration of 3-indoxyl sulphate continued to increase during and after pregnancy. Additionally, many pregnancy-associated metabolites were implicated in human diseases, especially inflammatory-related diseases (systemic lupus erythematosus and rheumatoid arthritis) and neonatal intrahepatic cholestasis based on a metabolite set library of disease signatures (Figure 4H). Some metabolite concentrations changed in both serum and faeces, but the trends were conflicting, suggesting a coordinated adjustment to support adaptation to the physiological state of pregnancy

Considering the role of SCFAs in physiological processes, targeted metabolomics analysis was performed to determine the dynamics of SCFAs during and after pregnancy. However, no temporal changes in SCFA concentrations were observed with advancing gestation (Figure S3).

### 3.7. Correlations between Pregnancy-Related Microbiota and Faecal and Serum Metabolites Presenting Temporal Changes during Pregnancy

**Figure 5:**
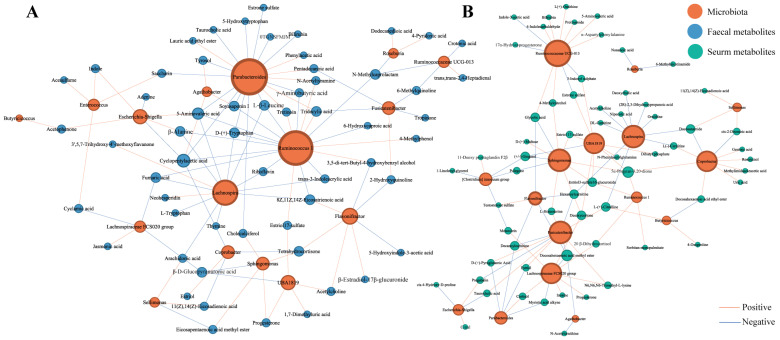
Interactions between pregnancy-related microbiota and metabolites. (**A**) Correlation network analysis of pregnancy-related microbiota and faecal metabolites. (**B**) Correlation network analysis of pregnancy-related microbiota and serum metabolites. Node size indicates the degree of corresponding factors. Only *p*-values < 0.05 are displayed in the network.

We performed Spearman’s correlation analysis to investigate the reciprocal interactions between pregnancy-related microbiota and faecal or serum metabolites presenting time-dependent changes during pregnancy (Figure 5). Notably, *Ruminococcus1* and *Ruminococcaceae UCG-013* were the main contributors to changes in faecal and serum metabolite concentrations, respectively. Changes in the concentrations of serum steroid hormones were strongly associated with variations in the microbiota. For example, the abundance of the *Lachnospiraceae FCS020 group* was positively correlated with the serum progesterone concentration, while the abundance of *Ruminococcaceae UCG-013* was inversely correlated with the concentration of 17α-hydroxyprogesterone. The abundances of *Ruminococcus1* and *Lachnospira* displayed inverse correlations with the faecal trans-3-indoleacrylic acid concentration, suggesting that pregnancy-related microbiota may influence tryptophan metabolism during pregnancy. Furthermore, the altered bacterial abundances influenced important neurotransmitters in the gut. For example, the abundance of *Flavonifractor* was strongly correlated with the faecal acetylcholine concentration. These findings suggest that pregnancy-related microbiota may contribute to the dynamic temporal regulation of metabolic changes.

## 4. Discussion

Pregnancy involves various physiological changes, including hormonal, immune, and metabolic shifts, which both influence and are influenced by the gut microbiome. Although the gut microbiota and associated metabolites have been recognised to considerably affect human health, our understanding of their dynamic changes and interactions during pregnancy and the postpartum period remains insufficient. Thus, in this study, we profiled the temporal dynamics of the gut microbiota and the faecal and serum metabolomes during and after pregnancy.

Our results demonstrated that the composition and signature shifted with advancing gestation. LEfSe analysis revealed unique characteristic bacteria during each trimester and the postpartum period, concentrated within genera belonging to the Lachnospiraceae or Ruminococcaceae families. In general, Lachnospiraceae represents one of the major taxonomic groups of the human intestinal microbiota [27]. Our results showed that genera associated with the Lachnospiraceae family, including the *Lachnospiraceae FCS020 group*, *Roseburia*, and *Agathobacter*, exhibited temporal changes as gestation progressed, with the abundance of the *Lachnospiraceae FCS020 group* increasing during pregnancy. Previous studies have linked members of Lachnospiraceae to obesity and diabetes, serving as biomarkers for prediabetes [28]. *Ruminococcaceae UCG-003* abundance was noted to significantly decrease with advancing gestation in our study. A previous human study showed that the abundance of *Ruminococcaceae UCG-003* is inversely correlated with cardiometabolic diseases and related symptoms [29]. Additionally, *Ruminococcaceae UCG-003* considerably affects host glucose homeostasis and lipid metabolism [30]. *Ruminococcus1*, as a major contributor to the gut microecosystem, is considered a favourable bacterium in obesity models [31,32,33], and its abundance increased steadily during pregnancy. From the perspective of host metabolism, two types of bacteria may play key roles during pregnancy. The first type, i.e., obesity-related bacteria (e.g., *Lachnospiraceae FCS020 group*), may favour energy and nutrient accumulation, ensuring a sustained supply of nutrition for the fetus and for the preparation of breastfeeding. The second type, referred to as obesity-friendly bacteria, may prevent excessive fat accumulation and associated pregnancy complications, such as gestational diabetes. This interplay between the two types of bacteria may serve as a long-term feedback mechanism for host metabolism and facilitate metabolic adaptation during pregnancy.

Additionally, the time-dependent alterations in the abovementioned bacteria during pregnancy may influence immune function. The depletion of *Ruminococcaceae UCG-003* is beneficial in inhibiting inflammatory responses that are harmful to the host [34]. Lachnospiraceae family bacteria can elicit the immune surveillance function of CD8+ T cells, thereby enhancing maternal immunity [35]. Given the complexity of immune modulation during pregnancy, which includes immunosuppression to accommodate fetal growth and the maintenance of robust immunity to protect against infection, it is speculated that time-dependent changes in bacterial abundance associated with immune responses may contribute substantially to immunological adaptations during pregnancy, but this warrants further research. Notably, although most pregnancy-associated bacteria within the Lachnospiraceae family are known for producing SCFAs, their levels did not significantly change over time during pregnancy. The function of Lachnospiraceae family bacteria appears to vary depending on the environment [35]. Thus, their primary function may not be the production of SCFAs in the highly hormonal intestinal environment during pregnancy, and they may regulate pregnancy functions through alternative metabolic pathways. For example, we found that the abundance of the *Lachnospiraceae FCS020 group* was positively correlated with the concentration of inosine, which is known to regulate energy metabolism and inflammatory responses [36]. These observations indicate that changes in the microbiota composition over time during pregnancy promote the healthy development of the fetus and prevent maternal illness.

After delivery, the abundance of several potential opportunistic pathogens, including *Escherichia_Shigella*, *Enterococcus*, and *UBA1819*, increased significantly compared with those during pregnancy. *Escherichia_Shigella* and *UBA1819* are known to be pro-inflammatory bacteria with increased abundances in various inflammatory disease conditions, such as acute liver injury [37] and colitis [38]. *Enterococcus* is known to express metalloproteases, thereby impairing the gut barrier and transmitting into the bloodstream in vulnerable hosts, resulting in inflammatory reactions [39]. These findings suggest a potential inflammatory response in women during the first week of the postpartum period. However, our study did not include continued follow-up beyond 1 week postpartum. Thus, the time required for the gut microbiome to return to the prenatal status remains unclear. Future research with extended postpartum follow-up may offer insights into future childbirth intervals from the perspective of the gut microbiota.

In addition to the gut microbiota, both serum and faecal metabolites exhibited temporal alterations over the course of normal pregnancy. Differential metabolites in serum and faecal samples were mainly involved in three metabolic pathways: steroid hormone biosynthesis, fatty acid metabolism, and amino acid metabolism (especially tryptophan metabolism). In the metabolic pathways related to steroid hormone biosynthesis, most pregnancy-associated serum steroids, especially progesterone and 17α-hydroxyprogesterone, increased in concentrations during pregnancy and rapidly returned to pre-pregnancy concentrations after delivery, which is consistent with the findings of previous studies [13,14]. 17α-hydroxyprogesterone is an important precursor in the biosynthesis of many steroid hormones, such as oestrogen, and a downstream metabolite of progesterone [40]. Reproductive hormones can modulate crucial physiological processes during embryonic development, which is crucial for maintaining pregnancy [41]. Recent publications have highlighted that the interaction between steroids and the gut microbiota is a bidirectional axis [42]. Some microorganisms can increase sex steroid levels through enterohepatic circulation [43], and Nuriel-Ohayon et al. found that progesterone modulates the pregnancy-associated gut microbial composition [44]. We found that the serum progesterone concentration was strongly positively correlated with the abundances of the *Lachnospiraceae FCS020 group* and *Sphingomonas,* which is consistent with a previous report [45]. Additionally, the abundance of *Ruminococcaceae UCG-013* was strongly correlated with the concentration of the serum 17α-hydroxyprogesterone level. Gut microbiota may interact with reproductive hormones to maintain physiological processes during pregnancy, while the specific mechanisms and pathways of microbial endocrinology in pregnancy remain unclear.

Furthermore, variations in the tryptophan–indole pathway during pregnancy were observed in both faeces and serum samples. Faecal trans-3-indoleacrylic acid concentrations gradually decreased from T1 to T2 and then increased continuously until the postpartum period. Indole derivatives can function as ligands for the aromatic meridian receptor (AhR) to influence the Th17 cell/regulatory T cell balance and immune function, leading to an improved immune response [46,47]. Furthermore, indole metabolites were directly transformed from tryptophan, which is completely dependent on the gut microbiota [48]. We found that the concentration of faecal trans-3-indoleacrylic acid was correlated with the abundance of specific bacteria (i.e., *Ruminococcus1*), suggesting that the pregnancy-related microbiota influences the tryptophan–indole pathway. These findings indicate that the gut microbiota may mediate temporal immune regulation according to the needs of different stages of pregnancy by affecting the production of specific indole derivatives, thus contributing to immune adaptation. The concentration of serum acetylcholine, a pivotal neurotransmitter for cognitive function [49], consistently reduced during pregnancy and increased soon after childbirth, attributable to its consumption to support the neural development of the fetus. However, the concentration of acetylcholine in faeces steadily increased during and after pregnancy. Previous studies have indicated that the gut microbiota can influence the central nervous system by synthesising neuroactive compounds or regulating the secretion of neurotransmitters, such as acetylcholine, to regulate cognition [50,51]. In this study, the abundance of *Flavonifractor* was positively correlated with the faecal acetylcholine level. This suggests that the gut microbiota may complement cognitive functions affected by fetal depletion by regulating neurotransmitter levels in the gut through the gut–brain axis. Thus, a highly coordinated microbiota–metabolite regulatory network may underlie the pregnancy process.

Fatty acids play a key role in supporting the rapid cell growth and activity in the developing fetus. Consistent with previous reports [13], the concentrations of certain serum fatty acids, such as 11(E)-eicosenoic acid and docosatrienoic acid, increased during pregnancy. However, the concentrations of several important polyunsaturated fatty acids in stool samples, especially eicosadienoic acid and 8Z,11Z,14Z-eicosatrienoic acid, gradually decreased during pregnancy and rapidly increased after childbirth, illustrating the consumption of maternal nutrients by the developing fetus. On the other hand, the gut microbiota can metabolize polyunsaturated fatty acids to confer host resistance to obesity [52]. Here, we found that the abundance of *Ruminococcus1* was negatively correlated with 8Z,11Z,14Z-eicosatrienoic acid levels. These observations suggest that the pregnancy-related microbiota may modulate unsaturated fatty acid metabolism to modify metabolic adaptation and the incidence of metabolic disorders during pregnancy.

Although longitudinal changes in maternal metabolites are beneficial for fetal development during pregnancy, some changes may be unfavourable to the mother. In addition to the depletion of important maternal fatty acids, such as γ-linolenic acid, an accumulation of indoxyl sulphate, which has pro-inflammatory properties, has been observed, possibly linked to changes in hepatic metabolism during pregnancy triggering the conversion of indole to excess indoxyl sulphate [53]. Alterations in the concentrations of these metabolites and elevated low-density lipoprotein concentrations are associated with an increased risk of cardiometabolic disease, which may explain prior evidence suggesting that multiple pregnancies may put women at higher risk of cardiovascular disease at an older age [54]. Collectively, these findings indicate the dynamic temporal regulation of microbial and metabolic changes during healthy pregnancy, contributing to maternal adaptation and fetal growth and development.

However, this study has several limitations. First, while high-throughput sequencing was performed in this study, a more comprehensive analysis necessitates high-resolution shotgun metagenomic sequencing. According to a previously published study, 30 patients in each group would likely be sufficient to assess phenotypic heterogeneity at the molecular level in a microbiota study [55,56]. Longitudinal sampling overcomes heterogeneity, which makes it possible to use small sample sizes in studies [57]. However, larger cohorts would potentially reveal additional microbe–metabolite relationships. In future studies, we will expand the sample size. Despite the strong correlations reported, causal inferences cannot be made conclusively. Further validation, such as investigating the immunological changes through transplantations of the faecal microbiota from pregnant and non-pregnant conventional mice into pregnant and non-pregnant germ-free mice, is necessary to confirm the role of the microbiota–metabolite network in driving maternal adaptation and fetal growth.

## 5. Conclusions

This study revealed temporal changes in the gut microbiota and faecal and serum metabolites during and after pregnancy through microbiota sequencing and untargeted metabolomics of faecal and serum samples. These findings highlighted that a highly coordinated microbiota–metabolite regulatory network may be involved in normal pregnancy, and this warrants further investigation. The insights derived from this work may provide guidance for health management during pregnancy.

## Figures and Tables

**Table 1:** Characteristics of participants during pregnancy.

Parameter (*n* = 31)	T1	T2	T3	*p* Value
Gestation weeks	10.9 ± 0.33	23.0 ± 0.47	35.5 ± 0.35	
Gestational weight gain (Kg)	1.39 ± 0.23	6.48 ± 0.71	12.39 ± 0.87	*p* < 0.0001
Cholesterol (mmol/L)	4.05 ± 0.14	5.97 ± 0.43	5.87 ± 0.34	*p* < 0.0001
Glucose (mmol/L)	4.49 ± 0.09	4.31 ± 0.12	4.39 ± 0.11	0.27
Triglyceride (mmol/L)	1.36 ± 0.09	3.21 ± 0.33	3.42 ± 0.35	*p* < 0.0001
High-density lipoprotein Cholesterol (mmol/L)	1.46 ± 0.07	1.69 ± 0.07	1.82 ± 0.17	*p* < 0.05
Low-density lipoprotein cholesterol (mmol/L)	2.07 ± 0.09	3.02 ± 0.35	3.08 ± 0.20	*p* < 0.0001
Cystatin C (mg/L)	0.67 ± 0.02	0.83 ± 0.05	0.92 ± 0.04	*p* < 0.0001
Total Protein (g/L)	69.24 ± 1.88	65.25 ± 0.54	63.59 ± 0.66	*p* < 0.0001
Alkaline phosphatase (μ/L)	45.70 ± 2.28	76.58 ± 4.51	120.80 ± 9.02	*p* < 0.0001

Data are presented as median (interquartile interval) or mean ± SEM. *p*-value based on the Mann–Whitney *U*-test or Student’s *t*-test, as appropriate. T1: first trimester; T2: second trimester; T3: third trimester.

## Data Availability

The data generated or analysed during this study are included in this article and its supplementary files. Other data that support the findings of this study are available from the corresponding authors upon reasonable request.

## Supplementary Materials

The following supporting information can be downloaded at https://www.mdpi.com/article/10.3390/nu16040483/s1. Table S1. Characteristics of participants; Figure S1. The composition of *Bifidobacterium* communities (A) and microbial functions altered during pregnancy (B). T1: first trimester; T2: second trimester; T3: third trimester; Post: postpartum; Figure S2. Quality control (QC) assessment for non-targeted metabolomics in faecal and serum samples. PCA score plots for all stool samples and QC samples in ESI^+^ (A) and ESI^−^ mode (B). PCA score plots for all serum samples and QC samples in ESI^+^ (C) and ESI^−^ mode (D). Spearman’s correlation analysis for faecal samples in ESI^+^ (E) and ESI^−^ (F) mode. Spearman’s correlation analysis for faecal samples in ESI^+^ (G) and ESI^−^ (H) mode; Figure S3. Temporal changes of short-chain fatty acids levels during pregnancy. (A) Acetic acid. (B) Propionic acid. (C) Isobutyric acid. (D) Butyric acid. (E) Isovaleric acid. (F) Valeric acid.
