# Supplementary material for: Dynamics of the Gut Microbiota and Faecal and Serum Metabolomes during Pregnancy—A Longitudinal Study
Source: Nutrients. 2024 Feb 7;16(4):483. doi: 10.3390/nu16040483 (PMC10892471; doi:10.3390/nu16040483)
Supplement: Supplementary file 1 [file nutrients-16-00483-s001.zip › nutrients-2815399-supplementary.pdf]

Supplementary Material

Table S1 Characteristics of participants

Parameter	Mean	SEM
Age	27.9	0.7084
BMI	21.71	0.5628
Parity	0.513	0.100
Spontaneous labor (n)	23	
Caesarean section (n)	8	

BMI: body mass index.

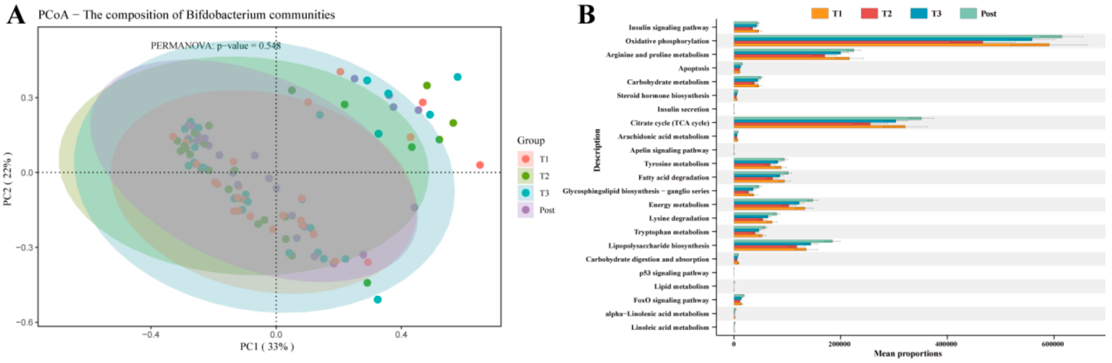

Figure S1 The composition of *Bifidobacterium* communities (**A**) and microbial functions altered during pregnancy (B). T1: first trimester; T2: second trimester; T3: third trimester; Post: postpartum.

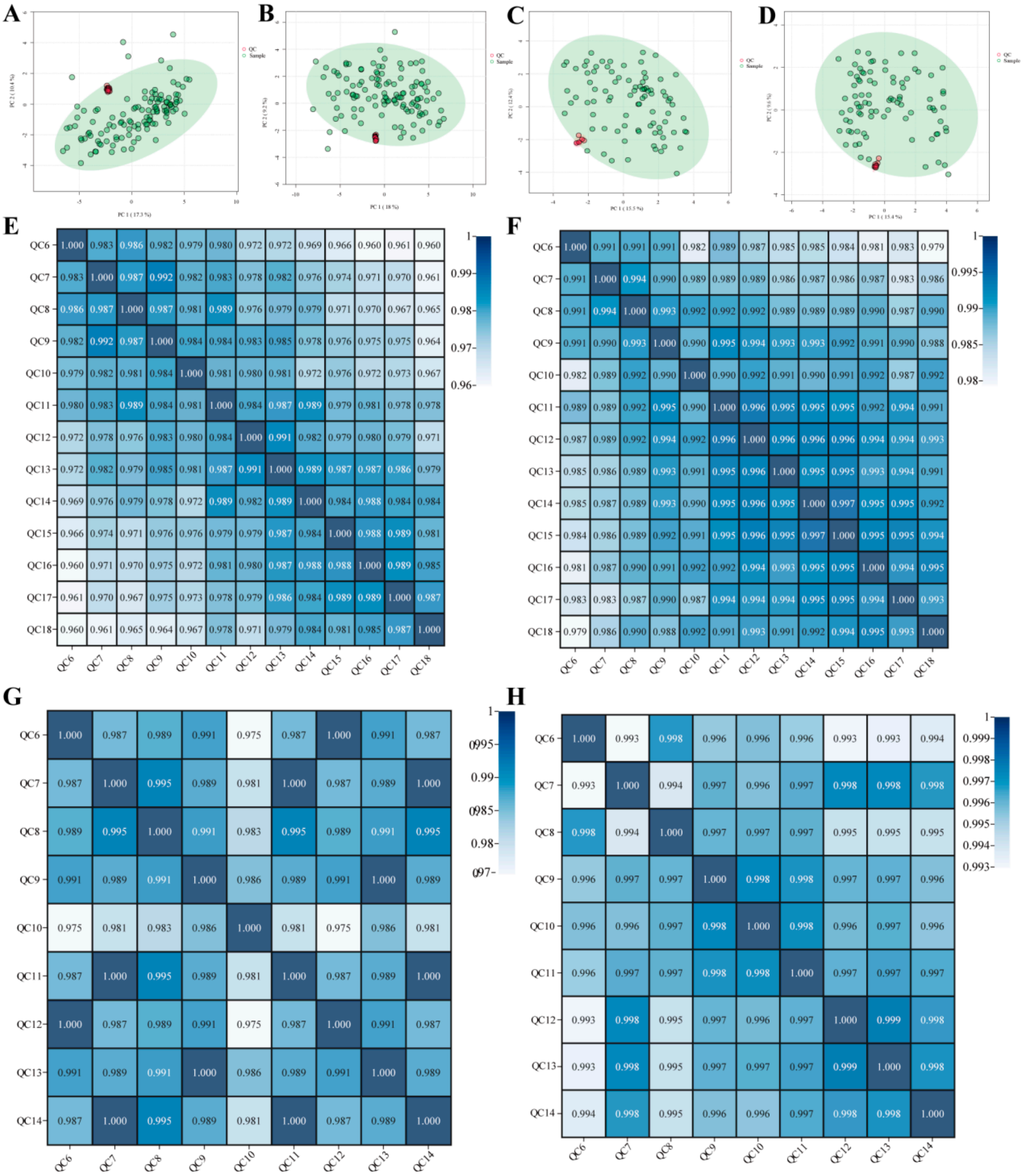

Figure S2 Quality control (QC) assessment for non-targeted metabolomics in faecal and serum samples. PCA score plots for all stool samples and QC samples in ESI$^+$ (A) and ESI$^-$ mode (B). PCA score plots for all serum samples and QC samples in ESI$^+$ (C) and ESI$^-$ mode (D). Spearman's correlation analysis for faecal samples in ESI$^+$ (E) and ESI$^-$ (F) mode. Spearman's correlation analysis for faecal samples in ESI$^+$ (G) and ESI$^-$ (H) mode.

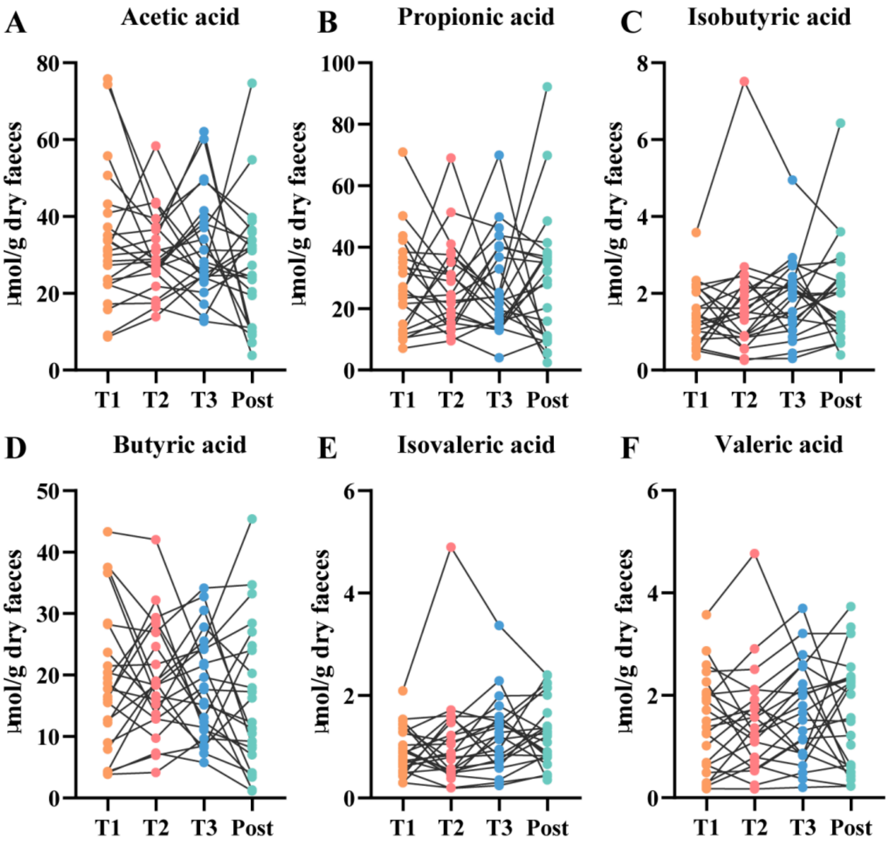

Figure S3 Temporal changes of short-chain fatty acids levels during pregnancy. (A) Acetic acid. (B) Propionic acid. (C) Isobutyric acid. (D) Butyric acid. (E) Isovaleric acid. (F) Valeric acid.
